# Reactions of physicians in the state of São Paulo to the use of telemedicine during the SARS-CoV-2 pandemic: cross-sectional study

**DOI:** 10.1590/1516-3180.2021.0475.R3.03112021

**Published:** 2022-04-11

**Authors:** José Luiz Gomes Amaral, Antonio Carlos Endrigo, Ana Maria Malik, Alberto José Niituma Ogata, Jorge Corrêa Assumpção

**Affiliations:** I MD, PhD. Full Professor, Discipline of Anesthesiology, Pain and Intensive Care, Department of Surgery, Universidade Federal de São Paulo (UNIFESP), São Paulo, Brazil.; II MD. Technology Director, Associação Paulista de Medicina (APM), São Paulo (SP), Brazil; and Chairman, Digital Health Committee, Associação Médica Brasileira (AMB), São Paulo (SP), Brazil.; III MD, PhD. Full Professor, Escola de Administração de São Paulo, Fundação Getulio Vargas (FGV EAESP), São Paulo (SP), Brazil.; IV MD, PhD. Researcher, Center for Health Planning and Management Studies, Escola de Administração de São Paulo da Fundação Getulio Vargas (FGV EAESP), São Paulo (SP), Brazil.; V MSc, MBA. Strategy and Marketing Superintendent, Associação Paulista de Medicina (APM), São Paulo (SP), Brazil.

**Keywords:** Telemedicine, Delivery of health care, Professional practice, COVID-19, Telehealth, Healthcare, Provision of health care, COVID-19 pandemic

## Abstract

**BACKGROUND:**

Telemedicine can be a component of integrated healthcare practices and its use is not a recent phenomenon around the world. In Brazil, its more widespread use began during the severe acute respiratory syndrome coronavirus 2 (SARS-CoV-2) pandemic, through extraordinary authorization from the Brazilian Ministry of Health.

**OBJECTIVES:**

To describe some aspects of use of teleconsultation among a sample of physicians in the state of São Paulo during the SARS-CoV-2 pandemic.

**DESIGN AND SETTING:**

Cross-sectional study based on a survey conducted by the São Paulo Medical Association (Associação Paulista de Medicina, APM) on medical practice during the SARS-CoV-2 pandemic between December 18, 2020, and January 18, 2021.

**RESULTS:**

This survey generated responses from 2,052 physicians. Of these, 981 (47.8%) reported not practicing any form of telemedicine. Among those who reported practicing telemedicine, 274 (28.4%) reported not receiving remuneration directly for the attendance provided and 225 (23.3%) reported receiving remuneration equal to what they would have received from face-to-face consultations. Regarding the professional linkage of the physicians who undertook telemedicine attendance, the majority (499; 51.8%) only attended private patients. Regarding the resources used to provide telemedicine attendance, most of the respondents used specialized digital platforms (594; 61.6%), electronic health records (592; 61.4%) and electronic prescriptions (700; 72.6%).

**CONCLUSION:**

This study demonstrates that important issues such as professional remuneration, use of electronic platforms and medical records, ensuring data protection and relationships between physicians and other stakeholders still need to be better defined, in order to achieve the desired scale and reach the outcomes defined.

## INTRODUCTION

Telemedicine can be defined as provision of healthcare in which the participants are separated in time and/or space, while telehealth is of broader nature, involving all health-related telecommunications applications.^1^ Telemedicine thus involves use of interactive information and telecommunications technologies, combined with computer systems, telemetry and biosensors to provide quality healthcare services that are not physically face-to-face and are outside the clinical-hospital space. It thus enhances the relationship between healthcare professionals and their patients, through eliminating geographical and time barriers.^2^

On the other hand, remote consultation can be defined as care provision mediated by technologies in which professionals and patients are in different physical spaces. It covers the same characteristic steps and responsibilities as in face-to-face attendance, including subjective, objective and diagnostic assessments, therapeutic proposals, requests for complementary tests, guidance and planning of care.^3^

The use of telemedicine is not a recent phenomenon: there have been reports of its use since the 1960s, but its more widespread utilization began with the development of the internet in the 1990s.^4^ For example, the American health plan and health provider Kaiser Permanente reported that in 2018, 47 million of the medical consultations they provided and 31 million prescriptions were issued online.^5^ The company Willis Towers Watson assessed cost effectiveness indicators in a study with the title “Current telemedicine technology can mean big savings,” published in 2014.^6^ The study suggested that telehealth had the potential to save more than $6 billion a year for companies in the United States. It constitutes an important tool within healthcare and there is evidence that it has an economic impact on national healthcare systems.^7,8^

Use of telemedicine as a component of integrated healthcare practices, such as preventive and chronic condition management programs, can be effective for clinical and administrative outcomes. Its attributes include greater availability of attendance, access to electronic medical records, online requesting of diagnostic tests, electronic prescription, availability of scientific material to support clinical decisions and reduction of the average duration of consultations. Moreover, as healthcare services become organized increasingly through integrated logic models, telemedicine ceases to be a support service and starts to have a cross-cutting role in all care.^9^

The Declaration of the 58^th^ General Assembly of the World Medical Association (WMA) in Copenhagen, Denmark, published in 2007 and amended by the 69^th^ WMA General Assembly, held in Iceland in 2018, defines telemedicine as remote practicing of medicine. Its interventions, diagnoses, treatment decisions and recommendations are based on data, documents and other information transmitted through telecommunication systems.^10^

With this definition as a reference point, remote practicing of medicine involving elaboration of diagnoses and treatments has generated intense debates within the medical profession. The promulgation of Resolution 2227/2019 of the Brazilian Federal Medical Medicine (Conselho Federal de Medicina, CFM), which extended and regulated the practices of telemedicine, can be highlighted.^11^ However, soon thereafter, this resolution was revoked at the request of regional medical councils, after complaints by both professionals and entities through various arguments (lack of debate and in-depth assessment of the subject, risks to patients, potential loss of jobs and/or precariousness of medical activity).

Although telemedicine has been discussed and used in healthcare systems for many years, including the adoption of technological innovations such as artificial intelligence, in Brazil it is still not fully used by healthcare professionals. However, the severe acute respiratory syndrome coronavirus 2 (SARS-CoV-2) pandemic has stimulated its use, particularly since the Ministry of Health published its Ordinance 467 on March 20, 2020, authorizing the use of teleconsultation.^12^ This enabled the continuation of a direct relationship between doctors and their patients, including for making diagnoses and defining treatments, during the healthcare crisis. Following this, law 13,989 was published by the Federal Congress,^13^ ratifying the Ministry of Health’s ordinance and also creating an environment for exchanges of medical documents such as prescriptions for medicines and legal attestations.

Use of telemedicine in the healthcare system involves adoption of technological resources, education of healthcare professionals and patients and integration into the healthcare system. Telemedicine attendance is considered to be a medical act, with all the technical and ethical implications involved in face-to-face consultation. In addition, telemedicine involves issues of data protection and patient privacy. In Brazil, the General Law on the Protection of Personal Data (Lei Geral de Proteção de Dados Pessoais, LGPD), published as Federal Law 13,709 in 2018, regulates how data can be collected and processed.^14^

On the other hand, there are some challenges, particularly in developing countries. A survey among Pakistani physicians concluded that evidence of effectiveness of telemedicine across different fields was inconsistent and lacked technical, legal, cultural and ethical considerations. Inadequate training, low levels of technological literacy and lack of infrastructure are the main barriers in implementing telehealth.^15^

A narrative review of factors influencing telehealth use across different medical specialties indicated that, while professional societies for specialties with lower telehealth use have played a limited role in providing guidance on telehealth use, their counterparts for the specialties with higher telehealth use have played a proactive role in advocating for consistent payment policies, developing guidelines for telehealth use, educating providers on getting started with telemedicine, advocating for telehealth training in medicine residency and developing resources for engaging patients in telehealth use. The review revealed that lack of reimbursement, lack of technology training and a ‘gatekeeper’ mindset could all serve as barriers to adoption of telehealth at the individual provider level. Hospitals and specialty societies could play an organized and proactive role in addressing each of these barriers through campaigning for better payment, promulgating guidelines for telehealth use, educating providers on how to get started with telehealth, promoting telehealth training in medical residency and engaging patients in telehealth services.^16^

On the other hand, a systematic review of outpatient telehealth implementation in the United States during the COVID-19 global pandemic identified three barriers impacting the implementation and use of telehealth resources: patient telehealth limitations, lack of telehealth guidelines for clinical care and issues relating to training, technology and finance.^17^

In the existing literature on telehealth, there has been consistent emphasis on the importance of recognizing the complexity of implementing telehealth services for successful and sustainable use. There are also multiple interdependent dimensions of telehealth to consider, including processes, user-experience and sustainability. Correspondingly, the design and implementation of telehealth services often involves engagement of stakeholders from a variety of disciplines, both within and outside the setting of the organization, including healthcare providers, managers, administrators, patients, information and communication technologists, economists and policymakers.^18^

With the rise of the COVID-19 pandemic, the course of telemedicine underwent a major upswing. As shelter-in-place became the norm around the world, patients and clinicians had to adapt to a new, yet not novel, way to provide medical care. Use of telemedicine will continue to grow in the post-pandemic world, but its development will depend on several factors. Some of those factors are related to patients, some to the physician and their practices and some to reimbursement.^15^

This article tries to fill the gap in the literature regarding the short-term reaction of physicians in the state of São Paulo, Brazil, to the use of telemedicine/teleconsultations after this procedure became officially approved in this country, due to the SARS-CoV-2 pandemic.

## OBJECTIVE

To describe some aspects of use of teleconsultation among a sample of physicians in the state of Sao Paulo during the SARS-CoV-2 pandemic.

## METHODS

This was a cross-sectional study based on a survey among doctors. It consisted of an analysis on responses to questions that formed part of a periodic survey conducted by the São Paulo Medical Association (Associação Paulista de Medicina, APM) on medical practice during the SARS-CoV-2 pandemic. Invitations to participate were sent out via electronic means (e-mails, social networks and websites) using the APM’s register of physicians, between December 18, 2020, and January 18, 2021.

In order to participate, potential respondents needed to firstly agree to the terms of a free and informed consent statement that they received. The present study was approved by the Ethics Compliance Committee on Research Involving Human Beings of the Getulio Vargas Foundation (Fundação Getulio Vargas, FGV), through opinion report no. 265/2020.

The variables analyzed in the present study were the following:

- Adoption of telemedicine (and its modalities)
- Remuneration of telemedicine attendance
- Linking of telemedicine attendance
- Technological resources used:
  d.1. Digital platform
  d.2. Electronic health record
  d.3. Electronic prescription
- Telemedicine training
- Patients perceptions of telemedicine

The data collected were compiled in the Microsoft Excel software, version 15.0, 2015 (Microsoft, Redmond, Washington, United States). These data were then tabulated for descriptive analysis, considering absolute and relative frequencies.

## RESULTS

This survey, which was available via electronic means from December 18, 2020, to January 18, 2021, generated responses from 2,052 physicians. It was sent out to a general mailing list of physicians containing 89,486 email addresses. The survey management system found that 4789 emails were opened and, from these, responses were received from 2,052 physicians. Among these physicians, 981 (47.8%) reported not practicing any form of telemedicine.

Among the physicians who reported practicing telemedicine, 274 (28.4%) reported that they had not received any remuneration directly for the attendance provided and 225 (23.3%) reported that they had received remuneration equal to what they would have received from face-to-face consultations. A further 149 physicians (15.5%) reported that they had received payment that was lower through the virtual procedure than what they would have received from face-to-face consultations. Lastly, 164 (17.0%) directly set the price of the consultation with the patient (Table 1). It is likely that many of these professionals started to provide care remotely, on a temporary basis, and thus did not develop a definitive remuneration model.

**Table 1.** Remuneration of physicians who attended telemedicine consultations

Type of remuneration	n	%	95% CI	
I did not receive remuneration for telemedicine attendance	274	28.4	25.6%	31.3%
I was paid per consultation, at the same rate as established for face-to-face consultations	225	23.3	20.7%	26.0%
I was paid per consultation, with an amount agreed jointly with the patient	164	17.0	14.6%	19.4%
I was paid per consultation, at a rate lower than that established for face-to-face consultations	149	15.5	13.2%	17.7%
I w I was paid per hour of work	147	15.2	13.0%	17.5%
I was paid per consultation, at a rate higher than that established for face-to-face consultations	5	0.5	0.1%	1.0%
**Total**	**964**	**100.0**		

CI = confidence interval.

Regarding the public attended through telemedicine, most of the respondents who used this model (630 physicians; 65.4%) attended both new and old patients. Most of the patients whom they attended did not have any complaints or evidence of SARS-CoV-2 infection (Table 2).

**Table 2.** Types of patients who received telemedicine attendance

Types of patients attended	Without SARS-CoV-2				With SARS-CoV-2			
	n	%	95% CI		n	%	95% CI	
Only old patients	281	29.1	26.3%	32.0%	53	5.5	4.1%	6.9%
Both new and old patients	452	46.9	43.7%	50.0%	178	18.5	16.0%	20.9%

SARS-CoV-2 = severe acute respiratory syndrome coronavirus 2; CI = confidence interval.

Regarding the professional linkage of the physicians who undertook telemedicine attendance, the majority (499; 51.8%) only attended private patients. Other physicians did this while working in medical-hospital institutions (188; 19.5%); or through participation in private healthcare insurance plans (231; 24.0%); or through an employment relationship with the organization in which they worked (46; 4.8%), as shown in Table 3. These findings highlight that many professionals started to remotely care for their former patients, on a temporary basis. With reorganization of care, after the emergency situation, this scenario will probably tend to change.

**Table 3.** Professional linkage of the physicians who undertook telemedicine attendance, according to the types of patients attended

Types of patients attended	n	%	95% CI	
Only private patients	499	51.8	48.6%	54.9%
Healthcare insurance plan beneficiaries	231	24.0	21.3%	26.7%
Patients at a medical-hospital care institution	188	19.5	17.0%	22.0%
Patients in organizations in which the physician was employed	46	4.8	3.4%	6.1%
**Total**	**964**	**100**		

CI = confidence interval.

Regarding the resources used to provide telemedicine attendance, most of the respondents used specialized digital platforms (594; 61.6%), electronic health records (592; 61.4%) and electronic prescriptions (700; 72.6%) (Table 4).

**Table 4.** Resources used by physicians for telemedicine attendance

Resources used	Yes, n (%)	95% CI		No, n (%)	95% CI	
Access to a specific digital platform for conducting teleconsultation	594 (61.6%)	58.5%	64.7%	370 (38.4%)	35.3%	41.5%
Remote attendance done with support from electronic medical records	592 (61.4%)	58.3%	64.5%	372 (38.6%)	35.5%	41.7%
Electronic prescriptions used	700 (72.6%)	69.8%	75.4%	264 (27.4%)	24.6%	30.2%

CI = confidence interval.

Regarding the experiences of patients who used the attendance provided through telemedicine, most of the physicians reported that the users accepted and liked the experience (788; 51.5%). However, 678 (44.3%) said that their patients accept this form of attendance only because of the SARS-CoV-2 pandemic but did not really like it. Another 64 (5.9%) said that their patients did not agree to use this resource.

Telehealth does not consist merely of transposition of face-to-face care to a virtual environment. It is permeated by actions of education, care, diagnosis and procedures.^11^ It requires training for proper and efficient use of the tools available. The present survey revealed that, out of the total number of respondents, 1,607 (88.33%) had not participated in any educational activities relating to telemedicine and that 27.97% (574) did not have any interest in participating in this in the future. Among the physicians who had participated in training activities, the majority had attended programs of duration less than four hours (238; 11.6%). In this context, the need for training professionals to provide care using the resources of telemedicine becomes relevant. Recently, a Brazilian guidebook for remote consultation was published and, certainly, other resources will be made available to Brazilian professionals.^3^

## DISCUSSION

Telemedicine has been widely used in several countries. Legal and regulatory issues still prevent it from advancing in Brazil. Its emergency use due to the SARS-CoV-2 pandemic has stimulated the entry of new service providers into the market and the use of information and communication technology (ICT) resources in a somewhat improvised way by professionals.

Telemedicine has the potential to increase the capacity for case resolution and facilitate coordination of care and therapeutic adherence. It can consequently reduce hospitalizations and unnecessary searches for emergency services. In terms of patient safety issues, its use during the pandemic can be considered to have constituted an appropriate use of resources, thereby reducing the misuse of face-to face consultations.

Although this use of telemedicine resources was an innovative experience for many of the physicians surveyed, the results from this study revealed that almost half of the respondents did not use telemedicine. In addition, more than a quarter of the participants who used it (28.4%) did not receive payment for the care they provided.

Telehealth needs to be part of an integrated care model, with action in a network. Telehealth should contribute to facilitating access to services, while maintaining coordination of care that is integrated with other dimensions and which gives due regard to the social determinants of health. In a recent study, the correlation between socioeconomic determinants and use of telemedicine services was measured and it was concluded that adoption of these services was significantly impacted by the social determinant factors of health, such as income, education level, race and insurance type.^18^

Lastly, incorporation of new technology within care, for example through use of remote consultations, brings the need to seek training for professionals regarding its use and its integration with secondary and tertiary-level healthcare services; and regarding adoption of safe processes, both for doctors and for patients. Training physicians to deliver high-quality, secure and personable healthcare through telemedicine can alleviate concerns and promote population-wide adoption of the technology. This is a key strategy that needs to be included in medical education and it is important to create opportunities for practitioners to learn more about this approach.^8^

It is important to build strategies and policies to enhance the use of telemedicine through deployment of appropriate infrastructure, continuous training and use of advanced technologies, with the aim of overcoming some pre-existing barriers and thereby ensuring high quality for professional medical actions.^18^

In a recent discussion about the telemedicine and current clinical practice trends during the COVID-19 pandemic, Wahezi et al. pointed out that adoption of telemedicine among physicians depends on reimbursement and on education to improve telemedicine consultations.^15^

Many physicians used telemedicine as support for their existing patients. It is known that there are more effective results when remote consultations are integrated into comprehensive healthcare.^2,8,9^ However, in the present study, many physicians who undertook telemedicine as a source of care did not use dedicated platforms (38.4%); nor did they use electronic health records (38.6%) or electronic prescriptions (27.4%). These resources are important for care to be provided in a more professional manner, so as to ensure the conditions for increased patient and provider safety, information integration and patients’ adherence to treatment.

### Limitations

The unquestionable limitation of this analysis relates to the sample analyzed: out of the total number of questionnaires distributed, only just over 2,000 were answered, which represents about 4% of the possible sample size. Moreover, even in this sample, only about 50% of the respondents said that they had been using telemedicine. This shows that although there was unclear risk of bias among the respondents, existence of this risk has to be recognized. In addition, the survey was applied only to physicians working in the state of São Paulo and is therefore not representative of the universe of Brazilian professionals.

## CONCLUSION

Currently, there is an international consensus that telemedicine is an important tool for medical practice that facilitates access to care, based on incorporation of new technologies and integration of the dimensions of prevention, diagnosis, treatment and monitoring. The SARS-CoV-2 pandemic has led to rapid adoption of telemedicine in various parts of the Brazilian healthcare system. However, as demonstrated in this study, important issues such as professional remuneration, use of electronic platforms and medical records, ensuring data protection and relationships between physicians and other stakeholders (healthcare insurance plans, hospitals and diagnostic centers) still need to be better defined, in order to achieve the desired scale and reach the outcomes defined.

Further research will be necessary with regard to the Brazilian scenario. There is a need to longitudinally assess different indicators relating to the efficiency of remote consultations and the perceptions of professionals and citizens.

This study has provided real-life knowledge of the general impressions and reactions of Brazilian physicians regarding telemedicine. This can improve the teaching of soft skills to medical and continuing education students, so as to impact their behavior as providers using the new technology. Managers may learn that physicians have concerns regarding adequate payment for use of these processes. Lastly, through telemedicine, the Brazilian population will increasingly be brought into the contemporary 21^st^ century way of practicing part of medical care, which is less time and effort-consuming.
